# A Guide to the Practical Study of the Diseases of the Eye

**Published:** 1859-11

**Authors:** 


ART. VII.—*A Guide to the Practical Study of the Diseases of the Eye: with an Outline of their Medical and Operative Treatment.* By JAMES DIXON, F.R.C.S., Surgeon to the Royal London Ophthalmic Hospital, Moorfield; formerly Assistant Surgeon to St. Thomas's Hospital. Second edition. Royal 12mo., pp. 431. John Churchill: London, 1859.

IT affords us much pleasure to be able to chronicle the appearance of a new edition of this work so soon after its original publication. The author must be greatly gratified with this evidence of the success of his literary labors, especially when we recollect the competitors which the work has in the treatises of Lawrence, Mackenzie and Jones, to say nothing of the numerous English monographs upon various subjects of ophthalmic medicine and surgery. The Review was among the first of the medical periodicals on this side of the Atlantic to point out the merits of Dr. Dixon's Guide, and to recommend it to the favorable notice of the American profession. An attentive perusal of it, soon after its publication, satisfied us that it was the most able manual on the diseases of the eye that had yet appeared in the English language; and a careful examination of the present edition has only served to corroborate that impression.

In preparing for the press a second edition of this work, the author has not considered it proper to amplify it by any considerable increase in the number of its pages; but has constantly kept in view his original plan of furnishing a practical manual on the subjects of which he treats. Some of the chapters have been re-arranged; and the section on the ophthalmoscopic appearances of the choroid and retina has been re-written, so as to render it more conformable to our present knowledge. The volume, like its predecessor, is gotten up in superior style, and reflects great credit both upon the author and publisher. We heartily wish that some competent person would enrich our literature with just such a production.

